# A suboptimal 5' splice site downstream of HIV-1 splice site A1 is required for unspliced viral mRNA accumulation and efficient virus replication

**DOI:** 10.1186/1742-4690-3-10

**Published:** 2006-02-03

**Authors:** Joshua M Madsen, C Martin Stoltzfus

**Affiliations:** 1 Interdisciplinary Program in Molecular Biology, University of Iowa, Iowa City, IA 52242, USA; 2 Department of Microbiology, University of Iowa, Iowa City, IA 52242, USA

## Abstract

**Background:**

Inefficient alternative splicing of the human immunodeficiency virus type 1(HIV-1) primary RNA transcript results in greater than half of all viral mRNA remaining unspliced. Regulation of HIV-1 alternative splicing occurs through the presence of suboptimal viral 5' and 3' splice sites (5' and 3'ss), which are positively regulated by exonic splicing enhancers (ESE) and negatively regulated by exonic splicing silencers (ESS) and intronic splicing silencers (ISS). We previously showed that splicing at HIV-1 3'ss A2 is repressed by ESSV and enhanced by the downstream 5'ss D3 signal. Disruption of ESSV results in increased *vpr *mRNA accumulation and exon 3 inclusion, decreased accumulation of unspliced viral mRNA, and decreased virus production.

**Results:**

Here we show that optimization of the 5'ss D2 signal results in increased splicing at the upstream 3'ss A1, increased inclusion of exon 2 into viral mRNA, decreased accumulation of unspliced viral mRNA, and decreased virus production. Virus production from the 5'ss D2 and ESSV mutants was rescued by transient expression of HIV-1 Gag and Pol. We further show that the increased inclusion of either exon 2 or 3 does not significantly affect the stability of viral mRNA but does result in an increase and decrease, respectively, in HIV-1 mRNA levels. The changes in viral mRNA levels directly correlate with changes in *tat *mRNA levels observed upon increased inclusion of exon 2 or 3.

**Conclusion:**

These results demonstrate that splicing at HIV-1 3'ss A1 is regulated by the strength of the downstream 5'ss signal and that suboptimal splicing at 3'ss A1 is necessary for virus replication. Furthermore, the replication defective phenotype resulting from increased splicing at 3'ss A1 is similar to the phenotype observed upon increased splicing at 3'ss A2. Further examination of the role of 5'ss D2 and D3 in the alternative splicing of 3'ss A1 and A2, respectively, is necessary to delineate a role for non-coding exon inclusion in HIV-1 replication.

## Background

The alternative splicing of retroviral mRNA is unique in that the inefficient splicing of viral precursor mRNA by the cellular splicing machinery results in the accumulation of unspliced mRNA which is necessary for the optimal expression of structural viral Gag, Gag-Pro, and Gag-Pro-Pol gene products. Approximately half of all HIV-1 mRNA remains unspliced; the remainder of the mRNA is either incompletely spliced, encoding the Env, Vpu, Vif, and Vpr gene products, or completely spliced, encoding the Tat, Rev, and Nef gene products.

**Figure 1:**
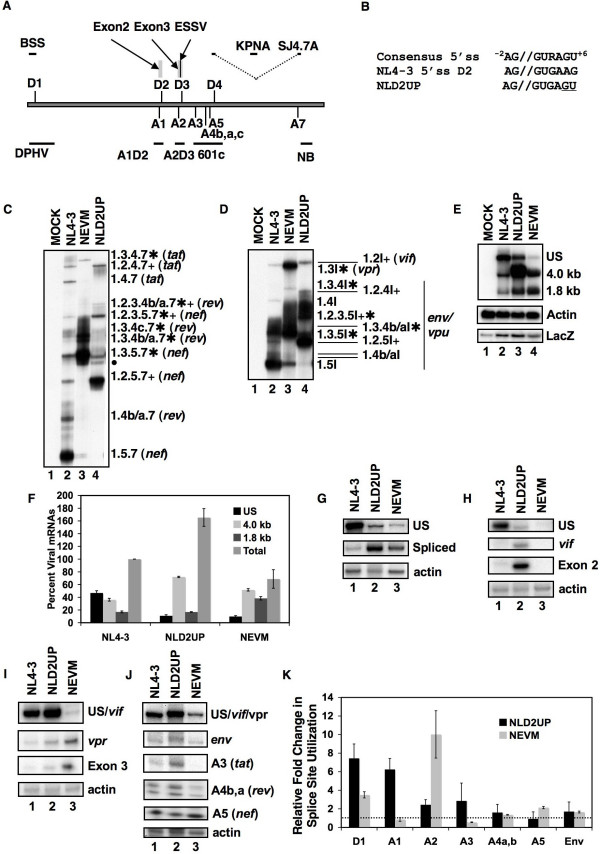
Inefficient inclusion of HIV-1 exon 2 is dependent upon a suboptimal signal at 5'ss D2. (A) Map of HIV-1 genome (NL4-3) showing the locations of 5' and 3' splice sites. The positions of Exon 2, Exon 3, and ESSV are indicated above the viral genome. Probes used to analyze HIV-1 splicing are shown above and below the viral genome and splice sites. Oligonucleotide primers used for RT-PCR analysis of viral splicing are shown above the viral genome. The BSS/SJ4.7A primer pair were used to detect the 1.8 kb, completely spliced viral mRNA species. The BSS/KPNA primer pair were used to detect the 4.0 kb incompletely spliced viral mRNA species. The probe complementary to the 3'-end of the viral mRNAs used for Northern analysis is indicated by NB. The probes used for the RNase protection assays (DPHV, A1D2, A2D3, and 601c) are represented by lines and are complementary to the splice sites to which they overlap. (B) 5'ss D2 within pNL4-3 was mutagenized as shown resulting in a consensus 5'ss signal in the infectious molecular clone NLD2UP. The previously described plasmid NEVM [15] was used as a control for increased splicing at 3'ss A2. Total RNA samples from Hela cells 48 hours post transfection with the indicated plasmids were analyzed by RT-PCR using primers specific for completely spliced viral mRNA (C) or incompletely spliced viral mRNA (D). HIV-1 RNA species are indicated on the right side of the gel by exon content, the mRNA to which they encode, and mRNA spliced at 3'ss A1 are indicated by plus signs and 3'ss A2 by asterisks. (E) Total cellular RNA from 293T cells 24 hours post transfection with the indicated plasmids was subjected to Northern blot analysis with a radiolabeled probe (NB) complementary to all HIV-1 mRNAs. (F) Northern blots were quantitated and the values shown were normalized to β-actin and β-galactosidase mRNA levels and represent the average of three independent experiments. RNA was also subjected to RPA analysis using the following riboprobes: DPHV (G), A1D2 (H), A2D3 (I), and 601c (J). Individual panels are representative of a single experiment. (K) Viral splice site utilization is represented relative to NL4-3 for each splice site. The values shown represent the average of three independents experiments and were normalized to β-actin and β-galactosidase mRNA levels.

Greater than 40 unique, alternatively spliced viral mRNAs are spliced within an HIV-1 infected cell by utilization of four viral donor splice sites (5'ss) and eight viral acceptor splice sites (3'ss) [1,2] (Fig. 1A). Regulation of HIV-1 alternative splicing occurs primarily because of the presence of suboptimal 5'ss and 3'ss, which decrease the recognition by the cellular splicing machinery of the splice signals [3-5]. Splicing at the viral splice sites is further regulated by the presence of exonic splicing enhancers (ESE) [6-10] and exonic/intronic splicing silencers (ESS/ISS) [6,9,11-14], which bind cellular factors and either promote or inhibit, respectively, splicing at neighboring splice sites.

Splicing at HIV-1 3'ss A2 results in the accumulation of *vpr *mRNA and inclusion of non-coding exon 3 when 3'ss A2 is spliced to the downstream 5'ss D3. We have previously shown that mutations which either disrupt an ESS within exon 3 (ESSV) or optimize the 5'ss D3 splicing signal, result in increased splicing at HIV-1 3'ss A2 [12,15]. Furthermore, increased splicing at HIV-1 3'ss A2 results in decreased unspliced mRNA accumulation and a reduction in virus replication, which was restored by second site reversions that either inactivate 3'ss A2 or 5'ss D3 [15].

In this report we have extended our analysis of HIV-1 alternative splicing by examining the effect on viral replication of increased splicing at HIV-1 3'ss A1. Increased splicing at 3'ss A1 results in the accumulation of *vif *mRNA and increased inclusion of exon 2 within spliced viral mRNA species. Our data show that a suboptimal 5'ss signal downstream of HIV-1 3'ss A1 is necessary for appropriate 3'ss utilization, accumulation of unspliced viral mRNA, Gag protein expression, and efficient virus production.

## Results

### Optimization of HIV-1 5'ss D2 results in increased splicing at 3'ss A1 and increased inclusion of exon 2

We have previously shown that disruption of ESSV within exon 3 results in increased splicing at 3'ss A2 and decreased unspliced mRNA accumulation. The excessive splicing phenotype was reversed by disruption of splicing at 5'ss D3 [15]. Conversely, mutations within 5'ss D3 that improve the 5'ss signal have been shown to increase splicing at 3'ss A2 [12]. To date, no cis-acting regulatory elements within exon 2 have been identified. Thus, in an effort to analyze the effect on HIV-1 replication of increased splicing at HIV-1 3'ss A1, we generated mutations within the downstream 5'ss D2 (NLD2UP) intended to increase the sequence homology to the metazoan 5'ss signal (Fig. 1B).

RT-PCR analysis of NLD2UP-transfected cells revealed that optimization of the 5'ss D2 signal results in increased accumulation of spliced viral mRNA that had been spliced at HIV-1 3'ss A1. Within the 1.8 kb completely spliced viral mRNA, increased accumulation of *nef*, *rev*, and *tat *mRNA species containing exon 2 (1.2.5.7, 1.2.3.5.7, 1.2.3.4b/a.7, & 1.2.4.7) was observed in NLD2UP-transfected cells when compared to NL4-3-transfected cells (Fig. 1C, compare lanes 2 and 4). Similarly, within the 4.0 kb incompletely spliced viral mRNA, increased levels of *env*/*vpu *mRNA containing exon 2 (1.2.5I, 1.2.3.5I, & 1.2.4I) and *vif *mRNA (1.2I) were observed in NLD2UP-transfected cells compared to NL4-3-transfected cells (Fig. 1D, compare lanes 2 and 4). Furthermore, the increased splicing at HIV-1 3'ss A1 resulting from improvement of 5'ss D2 in NLD2UP-transfected cells was similar to the increased splicing at HIV-1 3'ss A2 that occurs when ESSV is disrupted in NEVM-transfected cells (Fig. 1C, lane 3, and Fig. 1D, lane 3).

Northern blot analysis of viral mRNA from NLD2UP-transfected cells revealed that the relative accumulation of unspliced viral mRNA was decreased relative to the total viral mRNA in cells transfected with either NLD2UP or NEVM. In contrast, approximately half of viral mRNA remains unspliced in cells transfected with NL4-3 (Fig. 1F). Furthermore, when the total level of viral mRNA was taken into account, the increase in the 4.0 kb viral mRNA species was greater than the increase in 1.8 kb viral mRNA species in NLD2UP-transfected cells, compared to NEVM-transfected cells where the 4.0 and 1.8 kb viral mRNA species increased to a similar extent (Fig. 1E AND 1F). In addition there was an approximately two-fold increase in the level of total viral mRNA in NLD2UP-transfected cells and an approximately 2-fold decrease in the level of total viral mRNA in NEVM-transfected cells compared to NL4-3-transfected cells (Fig. 1F). The decrease in total viral mRNA in NEVM-transfected is in agreement with our previous reported results [15].

To quantitatively measure changes in HIV-1 3'ss utilization, RNase protection assays were performed using riboprobes overlapping the viral splicing signals (Fig. 1A). The overall level of splicing, as determined by utilization of HIV-1 5'ss D1, increased by approximately seven-fold in NLD2UP-transfected cells and three-fold in NEVM-transfected cells compared to the level of splicing observed in NL4-3-transfected cells (Fig. 1G and 1K). When the sequence homology of 5'ss D2 was increased relative to the metazoan consensus 5'ss signal there was a six-fold increase in the utilization of HIV-1 3'ss A1 (*vif *mRNA and exon 2 inclusion) compared to the level of splicing in NL4-3-transfected cells, whereas there was little change in splicing at 3'ss A1 in NEVM-transfected cells (Fig. 1H & K). Disruption of ESSV increased splicing at 3'ss A2 by approximately ten-fold compared to NL4-3, and cells transfected with NLD2UP utilized 3'ss A2 approximately two-fold more efficiently than NL4-3-transfected cells (Fig. 1I and 1K). Only small differences in splicing at HIV-1 3'ss A4a, A4b, A5, and *env *mRNA accumulation were observed in cells transfected with NLD2UP or NEVM when compared to NL4-3-transfected cells. (Fig. 1J & K). Interestingly, cells transfected with NLD2UP utilized HIV-1 3'ss A3 about two-fold more efficiently and cells transfected with NEVM spliced 3'ss A3 two-fold less efficiently than NL4-3-transfected cells (Fig. 1J and 1K). Alterations observed in splicing at 3'ss A3 by RNase protection assay within NLD2UP and NEVM-transfected cells were consistent with the increased and decreased accumulation of *tat *mRNA containing exon 2 (1.2.4.7) or exon 3 (1.3.4.7), respectively, as measured by RT-PCR (Fig. 1C compare lanes 3 and 4). These results indicate that in addition to increased splicing at HIV-1 3'ss A1 upon improvement of 5'ss D2 and at 3'ss A2 upon disruption of ESSV, increased splicing at 3'ss A1 and A2 also led to either increased or reduced levels of *tat* mRNA containing exon 2 or exon 3, respectively.

### Increased splicing at 3'ss A1 disrupts virus production

**Figure 2:**
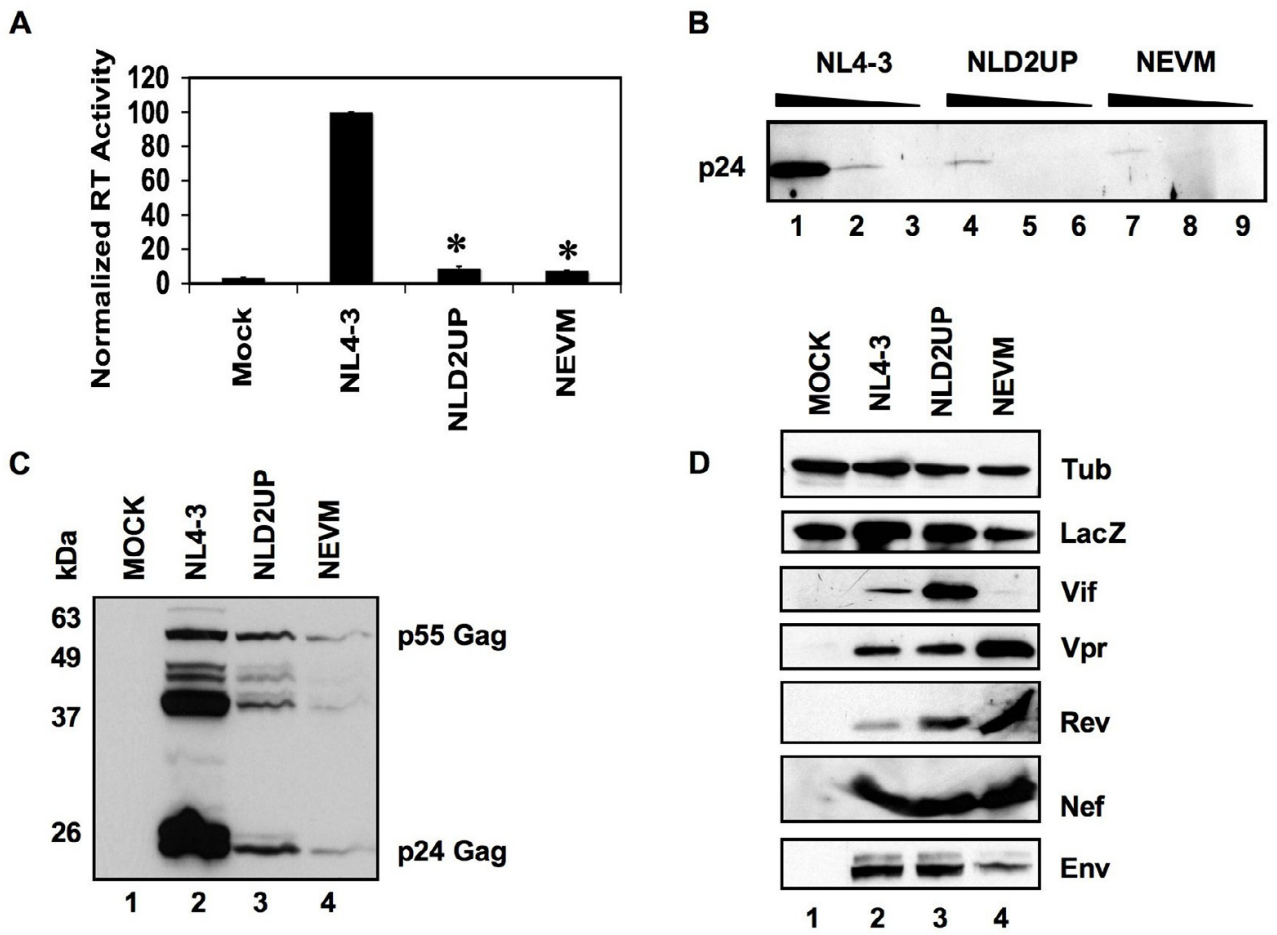
Efficient HIV-1 replication is dependent upon the presence of a suboptimal signal at 5'ss D2. (A) Reverse transcriptase activity of cell-free supernatants from 293T cells transfected with either NLD2UP or NEVM mutants. Asterisks indicate a significant difference when compared to mock transfected cells from three independent experiments (p < 0.01 by Student's t-test). (B) HIV-1 p24 Gag production from transfected 293T cells was measured by subjecting ten-fold serial dilutions of cell-free supernatants to Western blot analysis using serum from an HIV-1 infected patient. (C and D) Protein from transfected 293T cells was subjected to Western blot analysis using serum from an HIV-1 infected patient or antibodies to the indicated cellular or viral gene product.

Analysis of reverse transcriptase activity in cell-free supernatants from 293T cells that had been transiently transfected with NLD2UP resulted in an approximately ten-fold decrease in virus production when compared to pNL4-3-transfected cells (Fig. 2A). Furthermore, the greater than 90% reduction of virus production observed by mutagenesis of HIV-1 5'ss D2 was similar to the decrease observed when ESSV is mutated (Fig. 2A, NEVM). The ten-fold decrease in viral reverse transcriptase activity within the supernatants of NLD2UP-transfected cells correlated with an approximately ten-fold decrease in p24 Gag accumulation in cell-free supernatants as measured by Western blot analysis of serial dilutions of viral supernatants (Fig. 2B). As observed previously with the NEVM mutant, Gag accumulation was also decreased within 293T cells transiently transfected with NLD2UP as measured by Western blot analysis of cellular lysates (Fig. 2C).

To further characterize the defect in HIV-1 production upon mutagenesis of HIV-1 5'ss D2, HIV-1 structural, regulatory, and accessory protein expression was measured by Western blot analysis. Consistent with the mRNA analyses in Fig. 1G, 293T cells transiently transfected with NLD2UP expressed increased levels of HIV-1 Vif and cells transiently transfected with NEVM accumulated decreased levels of Vif when compared to wild-type NL4-3 (Fig. 2D). Also consistent with the mRNA analyses in Fig. 1H, Western blot analysis of HIV-1 Vpr expression in NEVM-transfected cells indicated increased levels of Vpr whereas cells transfected with NLD2UP expressed wild-type levels of Vpr. HIV-1 Rev, Nef, and Env expression within either NLD2UP or NEVM-transfected cells were at levels comparable to or somewhat greater than wild-type when normalized to levels of co-transfected β-galactosidase. Efforts to reproducibly detect Tat protein by Western blot were unsuccessful, and co-transfection of pCMV-Tat along with NEVM did not rescue the ability to produce wild-type levels of reverse transcriptase activity (data not shown). Based on the above data we concluded that optimization of 5'ss signal decreased the levels of cell-associated Gag and capacity to produce progeny virions to a similar extent as disruption of ESSV.

### Overexpression of an HIV-1 Gag-Pro-Pol plasmid rescues production of the 3'ss A1 and A2 oversplicing mutants

**Figure 3:**
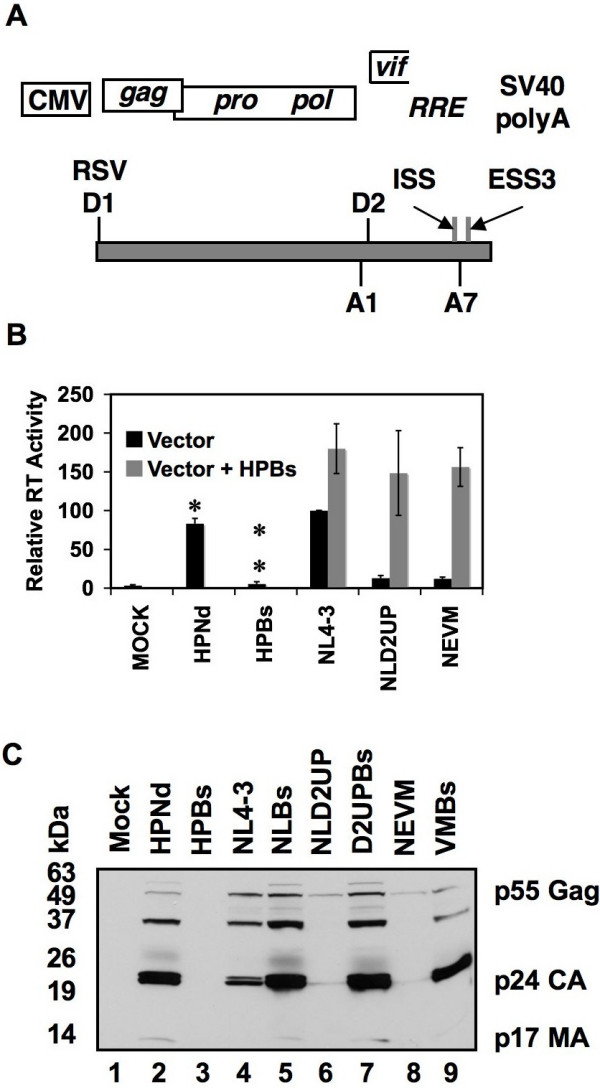
Restoration of HIV-1 virion production upon transient Gag, Gag-Pro, and Gag-Pro-Pol expression. (A) Open reading frames and signals contained within HPdBs. A representation of the HPdBs mRNA (grey box) is shown with the respective viral splice sites and splicing signals indicated. (B) Reverse transcriptase activity of cell-free supernatants from 293T cells transfected with the indicated plasmids, with or without the co-expression of the vector HPdBs. Black bars indicate the reverse transcriptase activity measured upon transient transfection of either HPNd, HPBs, or the indicated pNL4-3 derivative alone. Grey bars indicate the reverse transcriptase activity measured upon transient transfection of the indicated NL4-3 derivative along with HPBs. Reverse transcriptase activity represents the average of three independent experiments, normalized to the reverse transcriptase activity of supernatants from pNL4-3 transfected cells. Single asterisk indicates there is no significant difference when compared to NL4-3 transfected cells (p > 0.02 by Student's t-test) and double asterisk indicates there is no significant difference when compared to mock transfected cells (p > 0.2), from three independent experiments. (C) Protein from transfected 293T cells was subjected to Western blot analysis using serum from an HIV-1 infected patient. The HIV-1 Gag precursor (p55 Gag) and Gag proteolytic products (CA and MA) are indicated on the right.

The defect in HIV-1 virion production observed upon increased usage of either 3'ss A1 or A2 correlates with decreased expression of Gag. In order to confirm that decreased expression of HIV-1 Gag is responsible for the disruption of progeny virion production, an expression vector was generated that expressed HIV-1 Gag-Pro-Pol. The previously characterized retroviral packaging vector HPNd contains a nearly intact viral genome, with notable exceptions including the absence of the ψ RNA packaging signal and a deletion preventing Env expression [16]. HPNd is transcribed from the CMV promoter but because of the presence of the HIV-1 TAR and RRE, transcription of HPNd is still responsive to Tat expression and viral mRNA accumulation is still dependent upon Rev expression. HPNd was modified to minimize the potential of recombination with the 3'ss A1 and A2 oversplicing mutants, resulting in the vector HPBs (Fig. 3A). HPBs contains a deletion from just downstream of 5'ss D2, maintaining the entire Gag-Pro-Pol open reading frame, to just upstream of the RRE.

As expected because HPBs lacks the regulatory genes Tat and Rev, transient expression of HPNd but not HPBs in 293T cells resulted in near wild-type levels of reverse transcriptase activity in cell free supernatants (Fig. 3B). Furthermore, co-expression of HPBs with NLD2UP or NEVM restored reverse transcriptase activity to levels obtained when HPBs was coexpressed with NL4-3. Consistent with the restoration of reverse transcriptase activity upon co-expression of HPBs, the intracellular accumulation of p55 Gag and the p24 Capsid and p17 Matrix cleavage products were restored to wild-type levels in NLD2UP and NEVM-transfected cells by co-expression of HPBs, whereas cells transfected with HPBs alone did not express detectable levels of HIV-1 Gag (Fig. 3C). Rescued virion production after exogenous expression of Gag-Pro-Pol demonstrates that the primary defect in virus production in 3'ss A1 and A2 oversplicing mutants is the inability to accumulate sufficient quantities of unspliced viral mRNA and therefore express appropriate levels of Gag and Gag-Pro-Pol. Furthermore, since the transient Gag-Pro-Pol expression is Rev-dependent, it can be inferred from the complementation observed upon transient expression of Gag-Pro-Pol that sufficient quantities of Rev are expressed in 3'ss A1 and A2 oversplicing mutants. However, transient Gag-Pro-Pol expression, although responsive to Tat, is not dependent on Tat expression [17], therefore inferences about Tat expression from the 3'ss A1 and A2 oversplicing mutants cannot be made from these assays.

### Viral mRNA stability is not affected by non-coding exon inclusion

HIV-1 exon 2 and 3 have been suggested to play a role in viral mRNA stability, an observation that could possibly explain the disparity between the overall mRNA accumulation observed within NLD2UP and NEVM-transfected cells (Fig. 1E & F) [18]. In order to test whether or not non-coding exon inclusion influences spliced viral mRNA stability, we transfected 3'ss A1 and A2 oversplicing mutants NLD2UP or VMD3UP and analyzed spliced viral mRNA by RNase protection assays after treatment with Actinomycin D. In order to achieve maximal exon 3 inclusion, the vector VMD3UP was generated, which contains both the NEVM mutation and a previously described mutation within HIV-1 5'ss D3 that increases the affinity of 5'ss D3 with the metazoan 5'ss signal [12]. The double mutation further increases the inclusion of non-coding exon 3 when compared to either mutation alone. Inclusion of non-coding exons 2 or 3, respectively, in cells transfected with NLD2UP or VMD3UP was nearly complete within the spliced viral mRNA (Fig. 1C and 1D, and data not shown).

**Figure 4:**
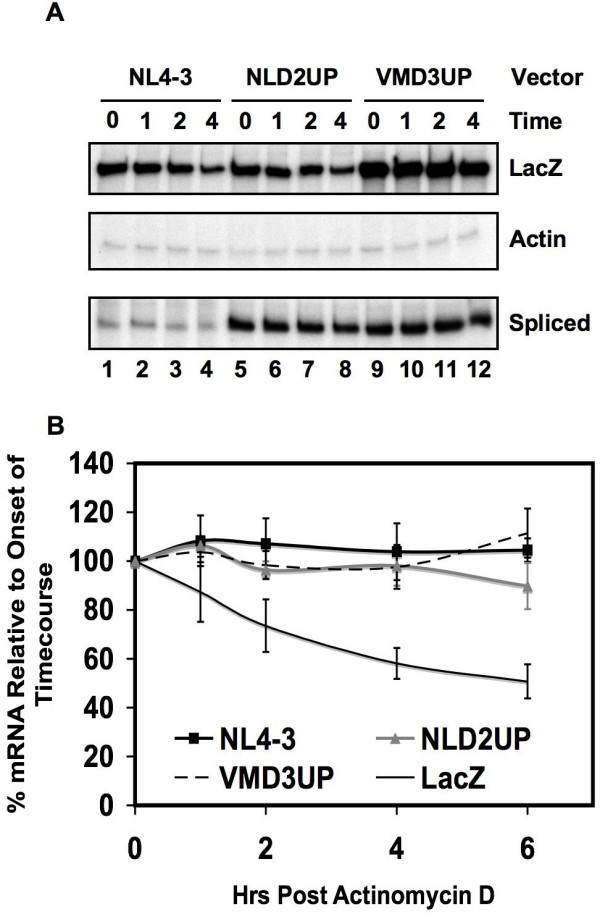
Effects of non-coding exon inclusion on viral mRNA stability. (A) RNase-protection mapping of HIV-1 spliced mRNAs using the DPHV riboprobe after transient transfection of the indicated plasmids and treatment with actinomycin D for the indicated times. Quantitation of the changes in accumulation of the spliced (B) viral mRNA species after the addition of actinomycin D (normalized to cellular β-actin) is shown relative to the onset of the experiment. The co-transfected pCMVβgal110 control was used to measure LacZ turnover, and the data shown represents LacZ mRNA levels within NL4-3 co-transfected cells. The data shown represent the average of three independent experiments.

The level of spliced viral mRNA remaining after 6 hours of treatment with actinomycin D did not differ whether inclusion of non-coding exon 2 (D2UP) was increased, or whether inclusion of non-coding exon 3 was increased (VMD3UP) (Fig. 4A and 4B). Further analysis of the spliced mRNA species by RNase protection assays revealed that there was no difference in the individual stabilities of the *env*, *tat*, *rev*, and *nef *spliced viral mRNA species upon inclusion of non-coding exon 2 and 3 (data not shown). Furthermore, the relative level of spliced viral mRNA remained stable throughout the experiment, when compared to the stability of the co-transfected β-galactosidase mRNA.

## Discussion

In this study we have extended our previous findings that HIV-1 virion production is disrupted upon increased splicing at HIV-1 3'ss A2 to show that increased splicing at HIV-1 3'ss A1 also disrupts virus production. Increased splicing at either 3'ss A1 or A2 results in a substantial decline in the relative level of unspliced viral mRNA resulting in decreased Gag protein expression. Two lines of evidence suggest that decreased Gag expression is the primary defect in virion production in the 3'ss A1 and A2 splicing mutants. First, expression of a Gag-Pro-Pol expression plasmid increased virus production in cells transfected with either the HIV-1 3'ss A1 or A2 oversplicing mutants to near wild type levels. These experiments strongly suggest that expression of Gag-Pro-Pol proteins is sufficient to rescue virus production, although from these experiments we cannot rule out the possibility that other RNA-mediated activities of the Gag-Pro-Pol expression plasmid may contribute to the rescue of virus production from the NLD2UP or NEVM mutants. Second, despite the greater than 90% decrease in particle production, p24 Gag accumulation was detected both intracellularly and extracellularly. This demonstrates that the inability to produce sufficient quantities of unspliced *gag *mRNA and not a defect in a downstream step in the virus life cycle is responsible for the replication defect in the 3'ss A1 or A2 oversplicing mutants.

Increasing the homology of the HIV-1 5'ss D2 to the metazoan consensus 5'ss signal dramatically increased the efficiency by which 3'ss A1 was spliced. Mutations that increase the homology of the 5'ss D3 also decreased unspliced viral mRNA accumulation and virion production but not as effectively as disrupting ESSV or increasing the sequence homology of 5'ss D2 (Madsen and Stoltzfus, preliminary data). The differences observed in the effect of increasing sequence homology of 5'ss D2 and D3 on unspliced viral mRNA accumulation and virion production suggests that HIV-1 exon 2 either contains no negative regulatory elements or a very weak ESS. We are currently testing for the presence of positive and negative splicing regulatory elements within HIV-1 exon 2.

The overall levels of viral mRNA were observed to increase or decrease in response to increased splicing at HIV-1 3'ss A1 and A2, respectively. Our studies indicated that the inclusion of either non-coding exon 2 or 3 has little or no effect on viral mRNA stability. The presence of non-coding exon 2 and 3 in viral mRNAs has been implicated in either the nuclear stabilization or degradation of the viral mRNAs in which they are present [18]. These previous studies analyzed the effect of non-coding exon inclusion on the stability of poly A+ RNA expressed from subgenomic viral constructs. In contrast, in our experiments the stability of total viral RNA that was expressed from intact viral genomes was analyzed.

Although the alterations in viral mRNA levels observed in response to increased splicing at HIV-1 3'ss A1 or A2 were not consistent with the previously described role of non-coding exon inclusion on viral mRNA stability, changes in *tat *mRNA levels correlated with the changes in the overall viral mRNA levels. Increased *tat *mRNA levels were observed when there was increased splicing at 3'ss A1 whereas decreased *tat *mRNA levels were observed when there was increased splicing at 3'ss A2. Differential *tat *mRNA accumulation would be expected to correlate with the respective change in viral transcription due to the ability of Tat to transactivate transcription from the viral LTR. Furthermore, it has previously been shown that HIV-1 non-coding exon 2 is included more frequently within *tat *mRNA species than non-coding exon 3. The difference in exon inclusion within the *tat *mRNA species is in contrast to the *rev *and *nef *mRNA species where exon 3 is preferentially included [2]. Taken together, these observations suggest that the difference in *tat *mRNA accumulation and the overall accumulation of viral mRNA in the HIV-1 3'ss A1 and A2 oversplicing mutants may be a consequence of more efficient splicing of 5'ss D2 to 3'ss A3 than 5'ss D3 to 3'ss A3.

Although not addressed in our studies, the increased splicing of viral mRNA in the HIV-1 3'ss A1 and A2 oversplicing mutants could result in the increased biogenesis of viral encoded miRNAs derived from spliced viral intron sequences. Recently, a viral encoded siRNA has been identified, corresponding to NL4-3 nt 7770–7788, located between HIV-1 5'ss D4 and 3'ss A7 [19]. The replication defects shown here did result in decreased unspliced viral mRNA accumulation relative to the total mRNA level which would be expected upon increased miRNA biogenesis. The viral encoded siRNA would be directed towards the incompletely spliced viral mRNA as well, which would also be expected to decrease. However, this was not the case, as shown in Fig. 1F. Furthermore, we showed that the stability of spliced viral mRNA species, which included incompletely spliced viral mRNA, did not decrease in response to increased viral splicing. These studies do not conclusively demonstrate whether or not increased viral miRNA biogenesis occurs upon increased viral splicing, since it has been shown that Tat abrogates the effect of miRNA expression [19].

If inclusion of non-coding exons 2 and 3 do not play a direct role in viral gene expression then why are these exons present in the viral genome? One possibility is that that the extent of inclusion or exclusion of exons 2 and 3 may be important in the maintenance of optimal levels of *vif *and *vpr *mRNAs under conditions during infection where the levels of cellular splicing factors are changing. An increase in negative factors binding to possible weak ESS elements within exon 2 and ESSV within exon 3, would decrease inclusion of these exons and thus, act to maintain the levels of the incompletely spliced *vif *and *vpr *mRNA. Conversely, an increase in positive factors binding to possible ESE elements within exons 2 and 3 would increase inclusion of these exons and act to prevent accumulation of excessive levels of *vif *and *vpr *mRNAs. A second possibility to explain the presence of exons 2 and 3 is that 5'ss D2 and D3 may be present to stabilize the incompletely spliced *vif *and *vpr *mRNAs by recruitment of U1 snRNP. A similar mechanism has been proposed for 5'ss D4, which has been shown to be necessary to stabilize HIV-1 *env *mRNA [8]. A third possibility is that 5'ss signals D2 and D3 may be necessary downstream of 3'ss A2 and A3, respectively, to optimize splicing efficiencies at these 3'ss and to attenuate the negative effects of ESS elements. In addition to playing a role in the recruitment of splicing machinery, 5'ss signals can recruit U1 snRNP in the absence of splicing at the 5'ss, thus activating splicing at the upstream 3'ss [20]. Further experiments to test the binding of U1 snRNP to 5'ss D2 and D3 in the presence and absence of splicing are required to test the role of 5'ss D2 and D3 in HIV-1 alternative splicing.

## Methods

### Plasmids

The infectious molecular clone pNL4-3 was obtained from the NIH AIDS Research and Reference Reagent Program [21]. pNLD2UP was derived by site-directed mutagenesis of pCMV5RIAG, which was generated by ligating the 2258 nt *EcoRI-AgeI* fragment of pNL4-3 into pCMV5 [15]. The resulting mutants were then digested with *EcoRI *and *AgeI *and ligated into pNL4-3. The following sense oligonucleotide was used to direct mutagenesis 5'ss D2, along with the complementary antisense oligonucleotide: ^5'^GGA CCA GCA AAG CTC CTC TGG AAA GGT GAG TGG GCA GTA GTA ATA CAA G^3'^. VMD3UP was generated by site-directed mutagenesis of pNEVM [15] with the previously described D3ATF primers [12]. The plasmid HPBs was derived from the vector pHP-dl. Nde/Ase or HPNd [16], by Klenow treatment followed by blunt-end ligation, after removal of the HIV-1 sequences corresponding to the 1746 nt *BsaBI*/*NdeI *fragment.

Riboprobe template constructs DPHV and 601c were generated by ligating the 884 nt *HindIII*/*PstI *and 601 nt *EcoRI*/*KpnI *fragments, respectively, of pNL4-3 into pBluescript SK+. The A1D2 and A2D3 riboprobe template constructs were generated by PCR amplification of pNL4-3 using the following oligonucleotide primers: A1D2 sense, ^5'^ATC GAA TTC AAA ATT TTC GGG TTT ATT ACA GGG^3'^, A1D2 antisense, ^5'^TGA AAG CTT TTC TTC TTG GCA CTA CTT TTA TGT CAC^3'^, A2D3 sense, ^5'^GTC GAA TTC AGT AGA CCC TGA CCT AGC^3'^, A2D3 antisense, ^5'^TCA AAG CTT AAC ACT AGG CAA AGG TGG^3'^. Amplification of viral DNA containing exon 2 or 3 was performed in 1 × AmpliTaq Gold, 0.5 μM each oligonucleotide primer, 0.05 ng Template DNA, 0.2 mM dNTP, 4.5 mM MgCl_2_, and 2.5 U AmpliTaq Gold Polymerase for 25 cycles of 30 sec at 95°C, 30 sec at 55°C, and 1 min at 72°C. Viral PCR products were digested with *EcoRI*/*HindIII *and ligated into Bluescript SK+. The pMapLacZ riboprobe template construct was used to analyze LacZ mRNA levels. The β-actin riboprobe template construct was generated by ligating the previously described β-actin PCR product [15] into pGEMT, using the pGEM?T Vector System II (Promega), according to the manufacturer's recommendations.

### Cells

293T and Hela cells were obtained from American Type Culture Collection, and were cultured as previously described [12]. For Gag overexpression experiments, 6 μg of HIV-1 plasmid was calcium phosphate precipitated with 6 μg of HP plasmid and 1 μg of pCMVβgal110 as previously described [12,15]. To measure viral mRNA turnover, 293T cells were plated at a density of 6 × 10^6 ^cells per 25 mL in a 15 cm dish 48 hours prior to transfection. Cultures were transfected with 75 μg DNA by calcium phosphate precipitation as described previously [12,15]. At 24 hours post transfection the cells were equally divided into five 60 mm dishes. Two hours after re-seeding, fresh media was added containing 10 μg/mL actinomycin D, and total RNA and protein was extracted at various times from 1 to 6 hr as previously described [22].

### Analysis of viral replication

In all experiments, 293T and Hela cells were transiently transfected with viral vectors as described previously [12,15], and viral replication was analyzed 24 hours post-transfection. Analysis of reverse-transcriptase activity, intra-cellular Gag expression, and viral accessory and regulatory protein expression has been described previously [15,23]. Western blot analysis was performed by using polyclonal antibody 2–37 directed against Rev [24] was used in immunoblotting at a dilution of 1:2000, polyclonal antibody 1–46 directed against Vpr (NIH AIDS Research and Reference Reagent Program) at a dilution of 1:500, and monoclonal antibody #319, directed against Vif (NIH AIDS Research and Reference Reagent Program) was used at a dilution of 1:50. Extracellular Gag was detected by performing ten-fold serial dilutions of cell-free supernatants in 0.04 M Tris, pH 6.8, 1% SDS, 10% glycerol, and 10% β-mercaptoethanol from transfected 293T cells, fractionating the diluted supernatants by SDS-PAGE, and performing immunoblotting as described previously for intracellular Gag [25].

### Analysis of HIV-1 splicing

Northern blot analysis of HIV-1 mRNA accumulation was performed as described previously [15]. The LacZ probe was generated by random-primed labeling of the 1443 nt *AvaI *fragment, digested from pCMV-110 β-galactosidase (β-gal) [26].

RNase protection assays were performed by incubating 1.6 × 10^6 ^cpm (HIV-1 probes) and 1.0 × 10^6 ^cpm (actin and lacZ probes), of *in vitro *transcribed, [α^32^P] UTP (Amersham) labeled RNA with 5 μg total cellular RNA. Radiolabeled probes were *in vitro *transcribed from linearized DNA templates as previously described [11], excluding the addition of a cap analog in the transcription reactions, with T3 RNA polymerase (DPHV and 601C probes) and T7 RNA polymerase (A1D2, A2D3, actin, and lacZ probes) (Stratagene). The samples were hybridized overnight at 57°C (A2D3 probe at 45°C) in a 35 μL reaction containing 40 mM 1 M PIPES pH 6.5, 400 mM NaCl, and 1 mM EDTA pH 8.0 in deionized formamide. RNase T1 (100 U) was added in 10 mM Tris pH 7.5, 5 mM EDTA pH 8.0, and 300 mM NaCl, and the samples were incubated for 30 minutes at 37°C. Fifty micrograms of Proteinase K was then added, SDS was added to a final concentration of 1.5% and the samples were incubated for 15 minutes at 37°C. The reaction  was extracted with phenol-chloroform, the RNA in the aqueous phase was  precipitated with ethanol, and the RNA was fractionated on a 5%  polyacrylamide gel containing 7M urea and 1/2X TBE at 500 V for 4 hours. The gels were analyzed by autoradiography, and the radiolabeled bands were quantitated using an Instant Imager (Packard).
